# Characterizing the microbiome recruited by the endangered plant *Firmiana danxiaensis* in phosphorus-deficient acidic soil

**DOI:** 10.3389/fmicb.2024.1439446

**Published:** 2025-01-15

**Authors:** Jiayu Li, Guangda Tang, Hongwei Liu, Xiaoying Luo, Juntao Wang

**Affiliations:** ^1^School of Tourism and Geography, School of Biology and Agriculture, Shaoguan University, Shaoguan, China; ^2^Hawkesbury Institute for the Environment, Western Sydney University, Penrith, NSW, Australia; ^3^College of Forestry and Landscape Architecture, South China Agricultural University, Guangzhou, China; ^4^Global Centre for Land-Based Innovation, Western Sydney University, Penrith, NSW, Australia; ^5^School of Science, Western Sydney University, Penrith, NSW, Australia

**Keywords:** plant microbiome recruitment, host selection, soil stoichiometry, endangered plants, rhizosphere microbiome, machine learning

## Abstract

Phosphorus (P)-deficient soils serve as crucial habitats for endangered plant species. Microbiomes play pivotal roles in soil element cycling and in determining a plant’s adaptability to the environment. However, the relationship between the endangered plant, microbiome, and soil stoichiometric traits, and how it affects plant adaption to P-deficient habitats remain largely unexplored. In this study, we investigated the microbiome (bacteria and fungi) in the rhizosphere of *Firmiana danxiaensis,* an endangered plant species growing exclusively in P-deficient acidic soils on Mt. Danxia, South China; the non-endangered coexisting tree species *Pinus massoniana was used as a reference.* Our results showed that soil traits in the rhizosphere of *F. danxiaensis* differed significantly from that of *P. massoniana*, including higher soil pH, lower C:N, and higher N:P. The rhizosphere of *F. danxiaensis* harbors higher microbial diversity and different microbial communities from *P. massoniana*. Using the machine learning approach, we characterized 76 bacterial and 20 fungal phylotypes dominated in *F. danxiaensis* rhizosphere, most of which had strong impacts on microbial ecological network structure (they accounted for only 0.33% node numbers but linked 21.2% of the nodes in the network); specifically, *Udaeobacter* spp., a highly abundant (constituting 4.07% of the total bacterial community) member of Verrucomicrobiota exclusively accumulated in the rhizosphere of *F. danxiaensis* but not *P. massoniana*, demonstrated a pronounced ecological prefers toward *F. danxiaensis* rhizosphere habitat (high pH, low C:N and high N:P) and potential antagonistic indication. In contrast, *P. massoniana* rhizosphere harbored more Subgroup2 of *Acidobacteria* and *Gammaproteobacterial* N-fixer. Taken together, this study provided novel evidence that endangered plants recruited a unique microbiome characterized by *Udaeobacter* spp. favoring high N habitat. It contributes not only to our understanding of microbiome recruitment by plants in P-deficient acidic soils, but also underscores the importance of microbiome in the conservation and population restoration of endangered plants.

## 1 Introduction

Soil carbon(C), nitrogen (N), and phosphorus (P) and soil stoichiometric characteristics are of significant importance for plant growth and ecosystem functioning (Delgado-Baquerizo et al., 2013). It has been revealed that endangered plants grossly persist under P-deficient soils at the global scale, while nitrogen–phosphorus deposition changed soil stoichiometric characteristics, posing a significant threat to endangered plants and endangering the global biodiversity scale (Wassen et al., 2005). Endangered plants are sensitive to the stoichiometric characteristics of soil, with a preference for high N:P ratios habitat (Lannes et al., 2012). Given that microorganisms exert significant influence on the biogeochemical cycling of C,N,P and thereafter influence soil stoichiometric characteristics (Zechmeister-Boltenstern et al.,2015; Epihov et al., 2021; Wang et al., 2022), unraveling the relationships between endangered plants, microbiome, and soil stoichiometric characteristics will provide valuable insights into the microbial mechanisms by which endangered plants adapt to P-deficient habitat (Lidbury et al., 2024). This will contribute to the development of strategies for the conservation and population recovery of endangered plant species.

Indeed, the soil microbiome is regarded as the ‘second genome’ of plants for its critical roles in plant colonization/local adaption via nutrition cycling, degrading harmful substances, and enhancing plant resistance (Panke-Buisse et al., 2015; Lau and Lennon, 2011). In detail, plants under stress can recruit beneficial microbial community through root exudates, mucilage, and sloughed cells from soil (Liu et al., 2019; Lakshmanan et al., 2014), also known as the ‘host selection’ process (Xiong et al., 2021); rhizosphere is considered the primary interface where plant recruits its microbiome. Increasing evidence suggests that microorganisms may play irreplaceable roles in the establishment and population maintenance of endangered plants. For instance, the utilization of arbuscular mycorrhizal fungi (AMF) inoculants has the potential to enhance *ex situ* conservation efforts and strengthen the success of reintroducing the endangered grass *S. umbrosus* (Zubek et al., 2009). Soil microbiome also contributed to the adaption of endangered grass *T. govanianum* along an elevational gradient in Mt. Himalayas (Islam et al., 2023). Bacteria/fungi selected might potentially contribute to the population maintenance of the endangered shrub plant *Helianthemum songaricum* (Xu et al., 2023). Although microbiome recruitment was predominantly driven by host plant species (Finkel et al., 2017), how plant–microbiome interaction contributes to the adaption of endangered plants to P-deficient habitats remains largely unexplored.

*Firmiana danxiaensis* H.H. Hsue and H.S. Kiu (Malvaceae) is an endangered species endemic to the P-deficient acidic soil in South China. Being a national Class II key protected wild plant in the Conservation Project for Plant with Extremely Small Populations (PESP) (Lu et al., 2019; Chen et al., 2014), *F. danxiaensis* has attracted growing interest due to its significant economic value and research potential (Lu and Luo, 2022; Chen et al., 2015; Li et al., 2024). This species is exclusively found in a few sites on Mt. Danxia, and study suggests that seed and pollen dispersal abilities might constrain its population development (Chen et al., 2024). However, whether and how plant–microbiome interaction affects the distribution of *F. danxiaensis* remains unknown. In this study, we used the high-throughput sequencing approach to investigate the microbiome in the rhizosphere of *F. danxiaensis*; *Pinus massoniana*, a common and fast-growing forest species coexisting with *F. danxiaensis*, was used as the contrast. We hypothesized that *F. danxiaensis* recruited microbiome that is accustomed to the soil stoichiometry (typically high N:P) in its rhizosphere, and these microorganisms constitute a key fundament for its adaption to the P-deficient environment. The aims of this study are to characterize the rhizosphere microbiome of *F. danxiaensis* and elucidate the microbial mechanisms supporting its establishment in P-deficient habitat, which will provide data support for developing conservation strategies of endangered plants, particularly in habitats impacted by global changes in N/P deposition.

## 2 Materials and methods

### 2.1 Field sites and sampling

A field survey was performed on two distantly separated populations of *F. danxiaensis*. One population is located in the Danxiashan National Nature Reserve (113°36′25″–113°47′53″E, 24°51′48″–25°04′12″N) with an area approximately 292 km^2^ (hereinafter referred to as Danxiashan) and the other one is located in Cangshizai County Nature Reserve (114°2′45″E, 25°08′19″N) with an area approximately 20 km^2^ (hereinafter referred to as Cangshizai). Both sampling locations are situated in Shaoguan City in northern Guangdong Province, South China, which is in the southern margin of the subtropics and is characterized by subtropical monsoon humid climate (Liu et al., 2018).

We set 4 sampling plots in Cangshizai and 12 sampling plots in Danxiashan, respectively. Given the limited distribution range of *F. danxiaensis*, our sampling has made an effort to cover all its distribution areas currently known. In each plot, five soil samples were randomly collected from the rhizosphere of *F. danxiaensis* and mixed to make a composite sample. In brief, we collected the intermediate and fine roots from the 0–15 cm soil depth, gently shook off the loosely attached non-rhizosphere soil, and then carefully brushed the rhizosphere soil (those tightly adhered to the roots) into ziplock bags. Rhizosphere soil of adjacent *P. massoniana* was collected as a contrast. Each soil sample was sieved through a 2 mm mesh and manually separated into two subsamples: One was stored at-80°C for microbial DNA sequencing, and the other was stored at 4°C for the analysis of soil physical and chemical properties.

### 2.2 Soil property measurement and DNA extraction

Soil properties were measured as previously described (Li et al., 2018). In brief, soil pH was measured using a pH meter at 1:2.5 soil: water ratio. Soil organic carbon (SOC) was measured using the K_2_Cr_2_O_7_ oxidation method. Soil total nitrogen (TN) and available nitrogen (AN) were determined by the Kjeldahl and the alkali solution diffusion method. Total phosphorus (TP) was measured by the alum molybdate yellow colorimetric method, and available phosphorus (AP) was measured by the Mo-Sb colorimetric method after immersion in 0.5 mol/L of NaHCO_3_. Available potassium (AK) was extracted using 1M ammonium acetate and measured on a flame photometer.

Soil metagenomic DNA was extracted from 0.25 g rhizosphere soil using a QIAGEN POWERSOIL DNA extraction kit. The concentration and quality of resultant extraction were measured by the NanoDrop One Microvolume UV-Vis Spectrophotometer (Thermo Fisher Scientific, MA, USA).

### 2.3 High-throughput sequencing and bioinformatic analysis

Soil bacterial and fungal communities were characterized by sequencing the 16S rRNA gene and Internal Transcribed Spacer (ITS) amplicons, respectively. Primer pairs 338F/806R (Srinivasan et al., 2012) and ITS5-1737F/ITS2-2043R (Bellemain et al., 2010; White et al., 1990) were used for 16S rDNA and ITS amplification, respectively. PCR was performed by following thermocycling: for initialization, 5 min at 94°C; 30 cycles of 30 s denaturation at 94°C, then annealing for 30 s at 52°C, and 30 s extension at 72°C; followed by 10 min final elongation at 72°C (Jiang et al., 2021). Resultant PCR products were sequenced with PE 250 bp strategy on the Illumina HiSeq 2500 platform (Guangdong Magigene Biotechnology Co., Ltd. Guangzhou, China).

We used a combined pipeline of USEARCH (Edgar, 2010) and QIIME2 (Bolyen et al., 2019) for bioinformatic analysis. In brief, pair-end reads were initially merged using USEARCH and then subjected to quality filtering, wherein the merged reads with an expected error (ee) set 1.0 maximum were retained. High-quality reads were dereplicated, and singletons were removed. Denoised sequences, referred to as zOTUs (with 100% sequence identity), were obtained by error correction of the amplicon reads using UNOISE3 (Edgar and Flyvbjerg, 2015). Representative sequences of 16S rDNA and ITS zOTUs (referred to as phylotypes afterward) were annotated against the Silva (Quast et al., 2012) and UNITE (Nilsson et al., 2018) databases within QIIME2, respectively. Approximately 2.1 M and 2.4 M high-quality merged sequences were mapped for bacteria and fungi, respectively. Prior to diversity calculations, a normalization procedure was applied, setting the count to 23,800 sequences *per* sample for both bacterial and fungal tables. Richness and Bray–Curtis (Bray and Curtis, 1957) matrices were calculated using the *vegan* package in R.^1^

### 2.4 Statistics

#### 2.4.1 Microbial feature identification with machine learning approach

We used a machine learning feature selection algorithm Boruta (Kursa and Rudnicki, 2010) to determine the featured microbial biomarkers in each tree species. Boruta is designed as a wrapper around the Random Forest classification algorithm. In Boruta, each microbial phylotype will compete with a randomly permuted version of the shadow feature; the feature can only be confirmed when it overtakes the best-randomized feature. Five hundred iterations were performed to minimize the uncertainty level. The feature selection was performed using the *Boruta* package in the R platform.

#### 2.4.2 Network analysis

We conducted co-existence network analyses to explore potential microbial interactions in the rhizosphere. Specifically, we identified bacterial and fungal phylotypes that showed significant correlations (positive or negative) in their relative abundance with others. To reduce the influence of rare taxa, we retained only those with more than 10 reads and 4 observations. We controlled the false discovery rate by performing 1,000 bootstraps using the FastSpar algorithm based on SparCC (Watts et al., 2019; Friedman and Alm, 2012). To optimize the network layout, we included only strong (*r* > 0.60) and robust (*p* =< 0.001) correlations in the final networks. The networks were visualized using the software Gephi (Bastian et al., 2009).

#### 2.4.3 Others

Tukey’s (Keselman and Rogan, 1977) honestly significant difference (HSD) test was used to examine the difference in rhizosphere soil property and stoichiometry characteristics between *F. danxiaensis* and *P. massoniana*. Linear and non-linear regression (Jarantow et al., 2023) fittings were used to elucidate the relationship between soil properties/stoichiometry characteristics and microbial diversity/featured phylotypes. Principal component analysis (PCA) was used to assess the variation of soil properties among sampling sites and tree species. Permutational multivariate analysis of variance (PERMANOVA) (Anderson, 2001) was conducted to assess the significance of differences in microbial communities between different groups. All these analyses were performed, and figures were generated in R using packages including vegan (Oksanen et al., 2012), ggplot2 (Wickham, 2016), dplyr (Wickham et al., 2023), and readxl (Wickham and Bryan, 2023).

## 3 Results

### 3.1 Soil property and stoichiometry

Soil properties analysis (Supplementary Figure S1) showed that there was no significant difference in SOM (3.9% vs. 2.8%) and TP (161 mg·kg^−1^ vs. 207 mg·kg^−1^) between *F. danxiaensis* and *P. massoniana* rhizosphere soils, but TN was significantly higher in *F. danxiaensis* rhizosphere soil (0.17%) than in *P. massoniana* rhizosphere soil (0.09%). *F. danxiaensis* rhizosphere soils also had higher pH, lower C:N, and higher N:P than *P. massoniana* rhizosphere soils, while no significant differences in C:P could be observed between them (Supplementary Figure S2). PCA and corresponding PERMANOVA revealed that the impact of tree species variation on rhizosphere soil properties is significant, while the impact of sampling locations was not significant (Supplementary Figure S3); there was no interaction between sampling points and species (*p* = 0.367).

### 3.2 Microbial community in the rhizosphere soils

A total of 7,573 bacterial and 2,279 fungal phylotypes were retrieved from all soil samples, respectively. The dominant phyla Acidobacteriota, Proteobacteria, Chloroflexi, Planctomycetota, and Verrucomicrobiota together composite the majority (81.8% relative abundance) of bacterial community (Supplementary Figure S4A), while dominant classes Agaricomycetes, Dothideomycetes, Eurotiomycetes, Leotiomycetes, and Sordariomycetes composite 85.7% of fungal community composition (Supplementary Figure S4B).

**Figure 1:**
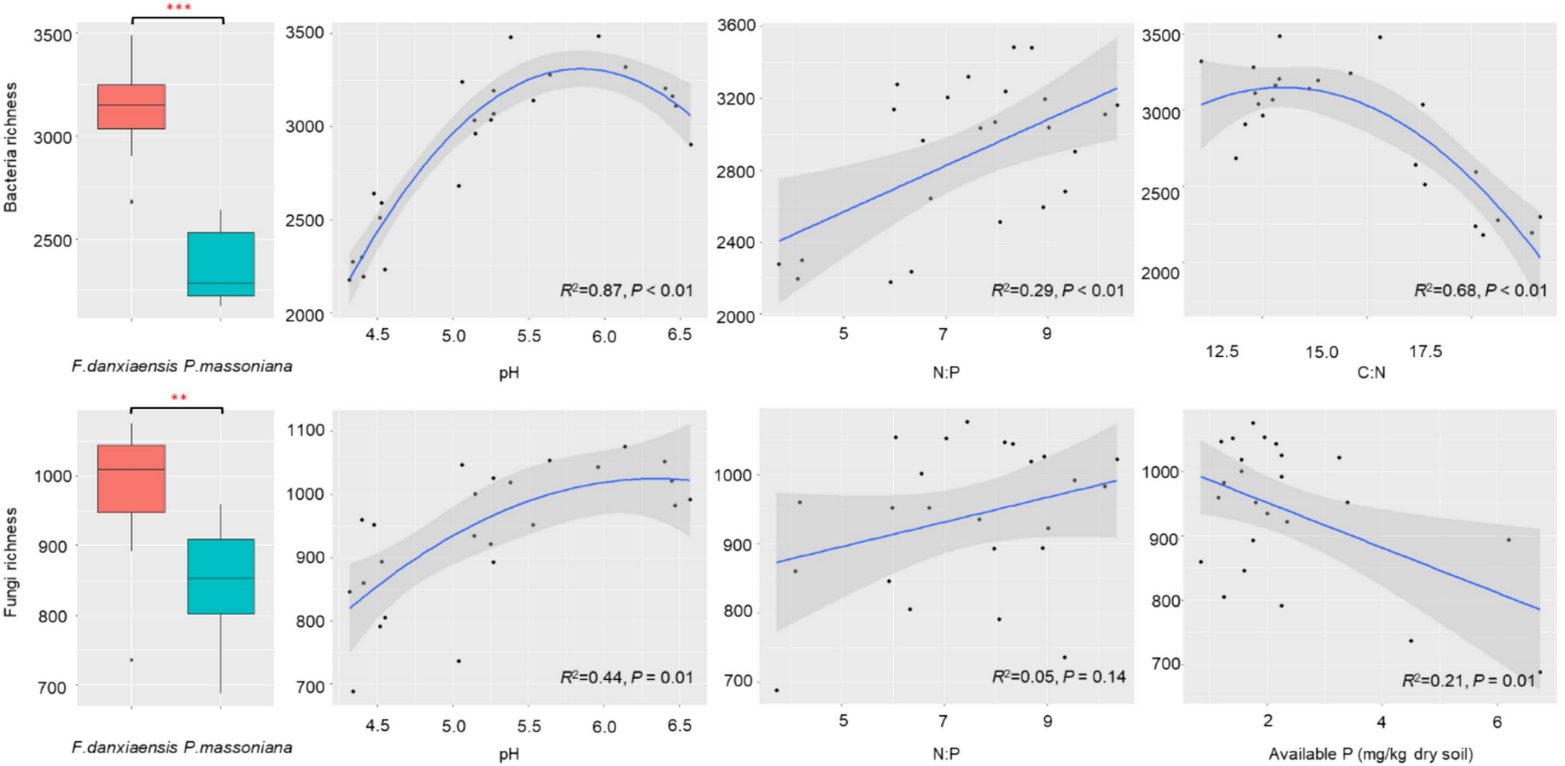
Soil bacterial and fungal richness in the rhizosphere of *F*. *danxiaensis* and *P. massoniana*, and their relationship with the significant predictors. Stars within each boxplot indicate a significant level (**p < 0.01, ***p < 0.001) according to Tukey’s HSD test. The shadow on the fitted curve in the scatter plots represents a 95% confidence interval. Significance of factors is determined as *per* the PERMANOVA analysis.

Both bacterial (*p* < 0.001) and fungal (*p* < 0.01) richness were significantly higher in *F. danxiaensis* rhizosphere than in *P. massoniana* rhizosphere (Figure 1). Factor analysis revealed that for both bacteria and fungi, pH is the predominant factor regulating their richness in the rhizosphere soils, followed by N:P (Table 1). While bacterial richness is also responsive to C:N (*p* = 0.025), fungal richness was otherwise significantly affected by-available P (*p* = 0.035). Regression models on the relationship between microbial richness and significant predictors (Figure 1) showed that bacteria are more strongly regulated by soil pH and N:P than fungi. Bacterial richness peaks at a pH of 5.8 and C:N of 14 and increases with increasing N:P, while fungal grossly increases with increasing pH and decreases with increasing available P.

**Table 1:** Permutational multivariate analysis of variance (PerMANOVA) on the factors predicting bacterial and fungal richness.

	Bacteria			Fungi		
	*R-sq*	*F*	*p*	*R-sq*	*F*	*p*
pH	0.575	51.32	0.001	0.415	21.06	0.002
C:N	0.088	7.81	0.025	0.003	0.14	0.745
C:P	0.022	2.00	0.186	0.001	0.05	0.847
N:P	0.083	7.40	0.016	0.119	6.02	0.025
Available P	0.004	0.38	0.555	0.109	5.53	0.035
Available N	0.007	0.61	0.467	0	0.01	0.951
Available K	0.042	3.70	0.066	0.038	1.92	0.185
Residual	0.179			0.315		
Total	1			1		

### 3.3 Featured microbial phylotypes in *F. Danxiaensis* and *P. massoniana* rhizosphere soils

**Figure 2:**
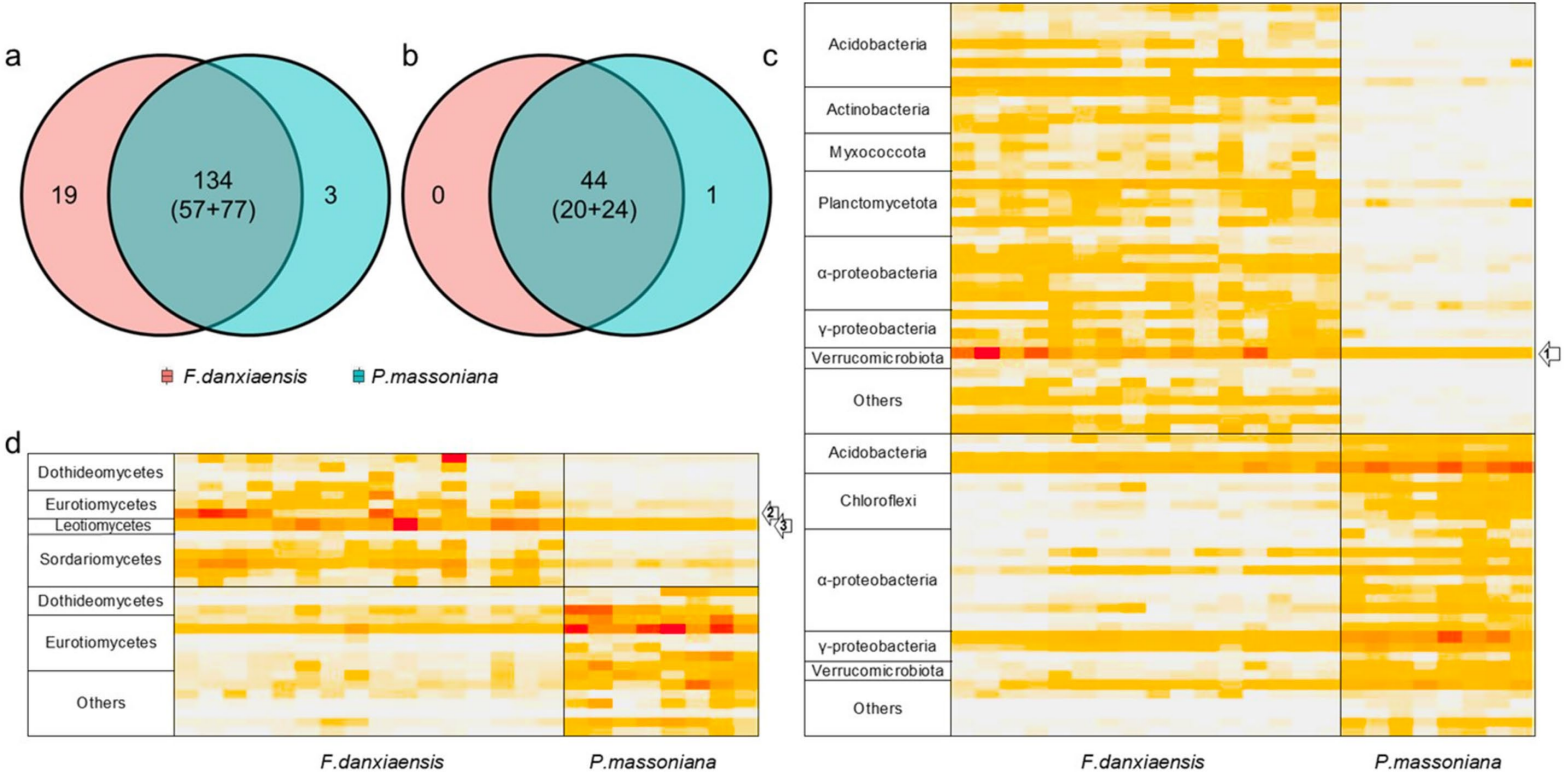
Featured bacterial and fungal phylotypes in the rhizosphere of *F*. *danxiaensis* and *P. massoniana.* Venn diagrams show the number of featured bacterial **(A)** /fungal **(B)** phylotypes for *F. danxiaensis* and *P. massoniana,* and numbers in brackets indicate those detected in both soils but be determined as the feature of one tree due to its much higher relative abundance than in the other, *e.g.*, 57 featured bacterial phylotypes for *F. danxiaensis* and 77 for *P. massoniana*. **(C)** Heatmap demonstrates the relative abundance of featured bacterial phylotypes detected. Grouped at the genus level and labeled at the phylum level for clarity. **(D)** Heatmap demonstrates the relative abundance of featured fungal phylotypes detected. Grouped at the genus level and labeled at the class level for clarity. Labels 1, 2, and 3 in the heatmap indicate dominant featured *phylotypes* accumulated in *F. danxiaensis* rhizosphere. 1 *Udaeobacter*; 2 uncultured *Pyrenulales*; and 3 *Leohumicola lenta*.

**Figure 3:**
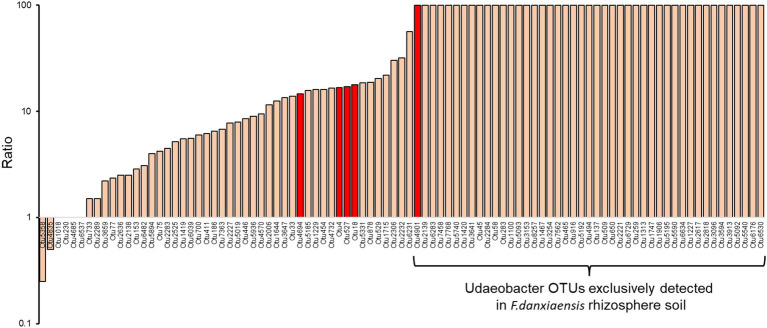
Ratio of Udaeobacter phylotypes relative abundance in *F. danxiaensis* rhizosphere soil to those in *P. massoniana* rhizosphere soil. Those in red were identified as featured phylotypes using the machine learning algorithm. The y axis is displaced on a logarithmic scale to help visualize the data by compressing extreme values and stretching out smaller ones. Those not detected in *P. massoniana* rhizosphere soil were assigned 100 to suit the log transformation.

Using the machine learning model, we identified 156 bacterial phylotypes (2.06% out of the overall 7,573) and 45 fungal phylotypes (1.97% out of the overall 2,279) that dominated in *F. danxiaensis* or *P. massoniana* rhizosphere (Figures 2A,B). For bacteria, 19 featured phylotypes were exclusively detected in *F. danxiaensis* and 3 were exclusively detected in *P. massoniana*, while only 1 fungal exclusively existed in *P. massoniana*. Eight of Planctomycetota phylotypes were exclusively detected in *F. danxiaensis*. Most of the microbial features can be detected in both trees but would have a much higher abundance in one tree species than in the other. Of them, *Udaeobacter* from the phyla Verrucomicrobiota, which accounted for 4.07% relative abundance of the whole bacterial community, was the most dominant bacteria that accumulated in the *F. danxiaensis* rhizosphere (Figure 2C). We further examined all the 93 *Udaeobacter* phylotypes (including the non-featured and featured ones determined by the machine learning model) and found that nearly all of them had much higher abundance in *F. danxiaensis* rhizosphere than in *P. massoniana* rhizosphere (Figure 3). Specifically, nearly half of them were exclusively detected in *F. danxiaensis* rhizosphere soil. In addition to *Udaeobacter*, two abundant fungal phylotypes, namely, unassigned *Pyrenulales* and *Leohumicola lenta*, were found to accumulate in *F. danxiaensis* rhizosphere. They had a total relative abundance of >2% in the fungal community.

By contrast, the most dominant microbial phylotypes featured in *P. massoniana* rhizosphere soils include one from subgroup2 of *Acidobacteria*, two nitrogen fixer *Burkholderia–Caballeronia–Paraburkholderia*, and *Phialomyces macrosporus* of Ascomycota. Together, they account for 2.30% of total bacterial abundance and 1.69% of total fungal abundance.

### 3.4 Ecological preference of top dominant microbial features

**Figure 4:**
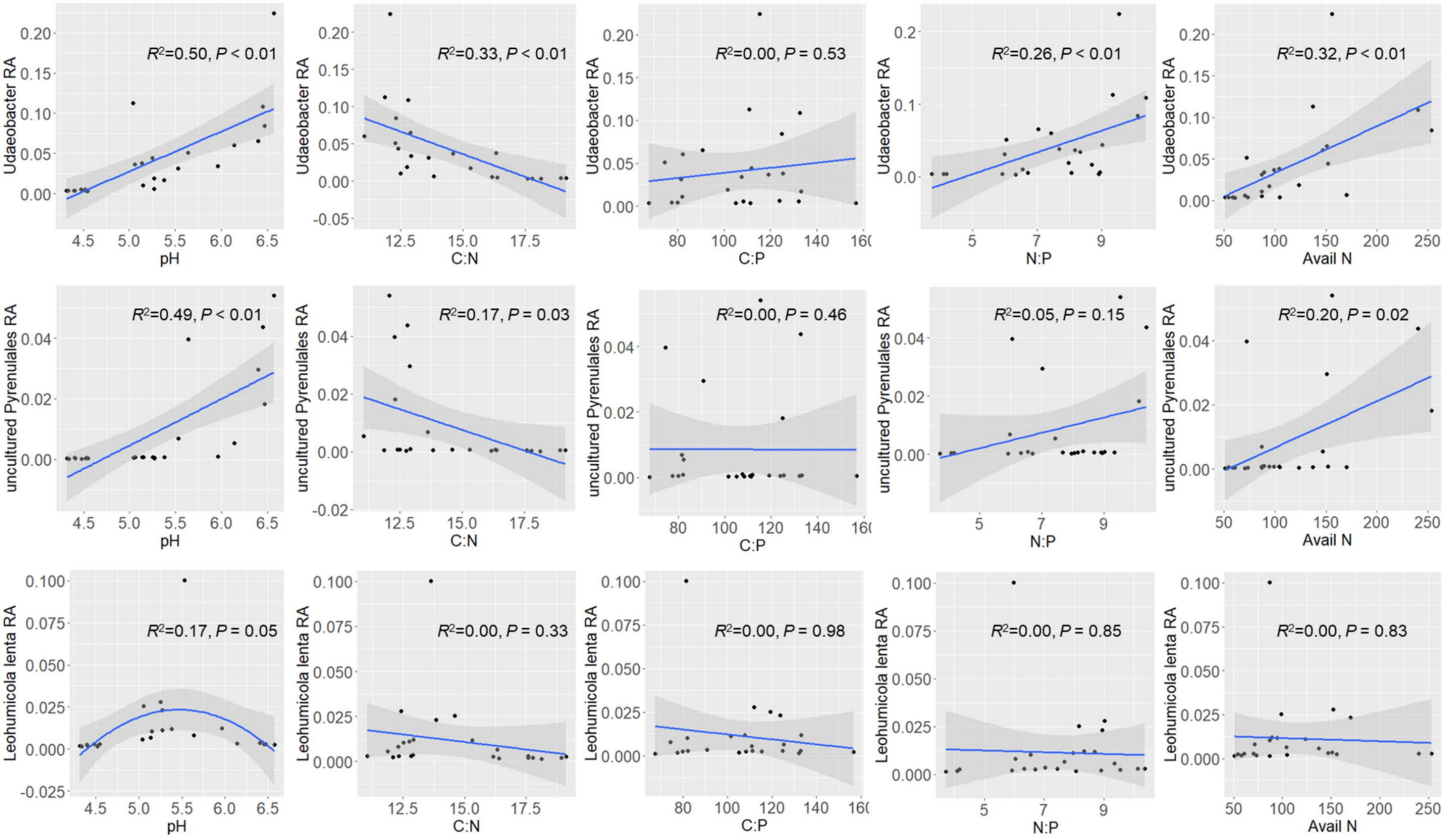
Ecological preference of dominant microbial features (*Udaeobacter*, uncultured *Pyrenulales*, and *Leohumicola lenta*). The shadow on the fitted curve in the scatter plots represents a 95% confidence interval. RA means relative abundance. Avail N means available nitrogen.

Regression analysis was performed to examine the relationship between soil property/stoichiometry characteristics and the top dominant featured microbial phylotypes (Figure 4). The relative abundance of *Udaeobacter* was found to increase with increasing soil pH. It decreased with an increase of C:N but increased with increasing N:P. Another phylotype unassigned *Pyrenulales* exhibited a similar but weaker pattern as *Udaeobacter*. *Leohumicola lenta* was only sensitive to soil pH, with a peak at pH 5.5, but had no significant relationship with any other stoichiometry characteristics.

### 3.5 Microbial co-existence network

**Figure 5:**
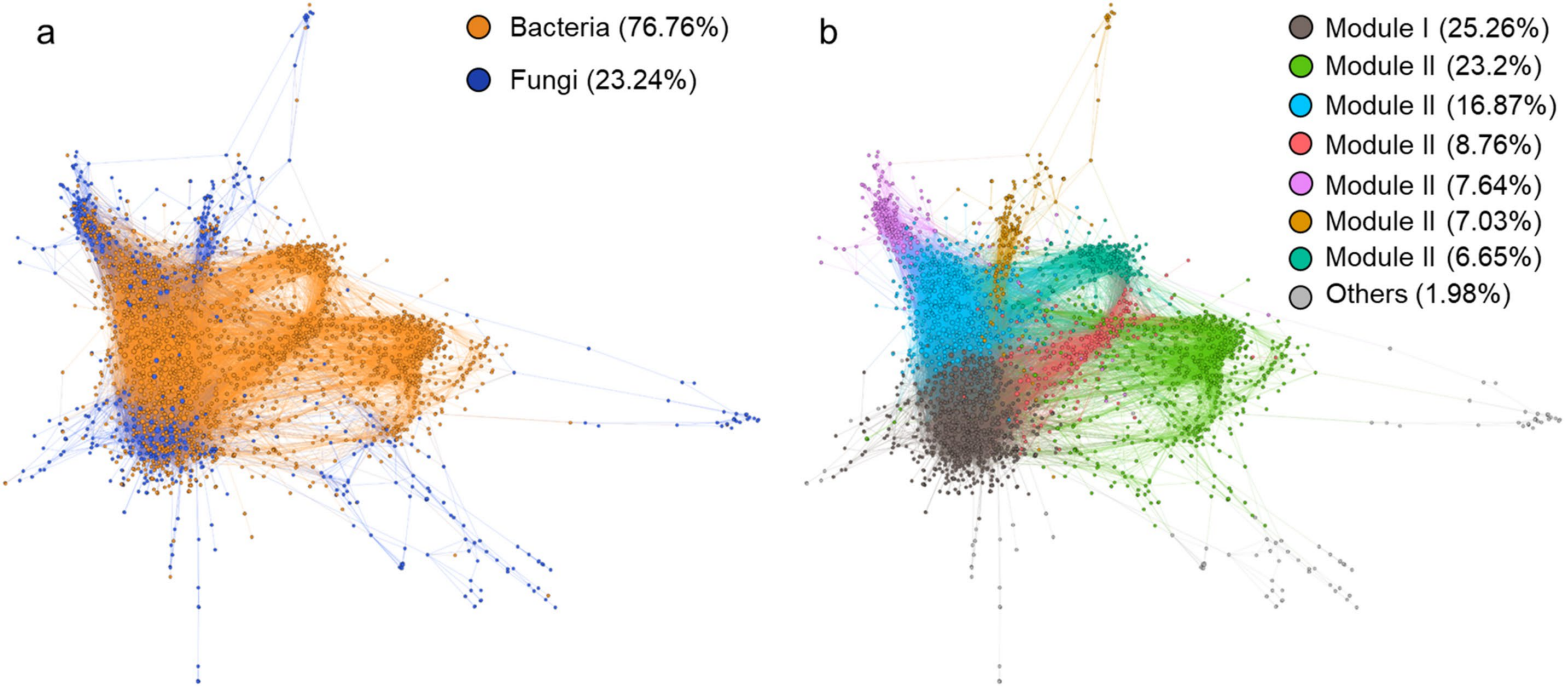
Co-existence network of soil bacteria and fungi in the rhizosphere. Nodes size is proportional to the degree, and edge width is proportional to the correlation strength. **(A)** Nodes are colored as *per* the kingdom, and **(B)** nodes are colored as *per* the modularity.

**Figure 6:**
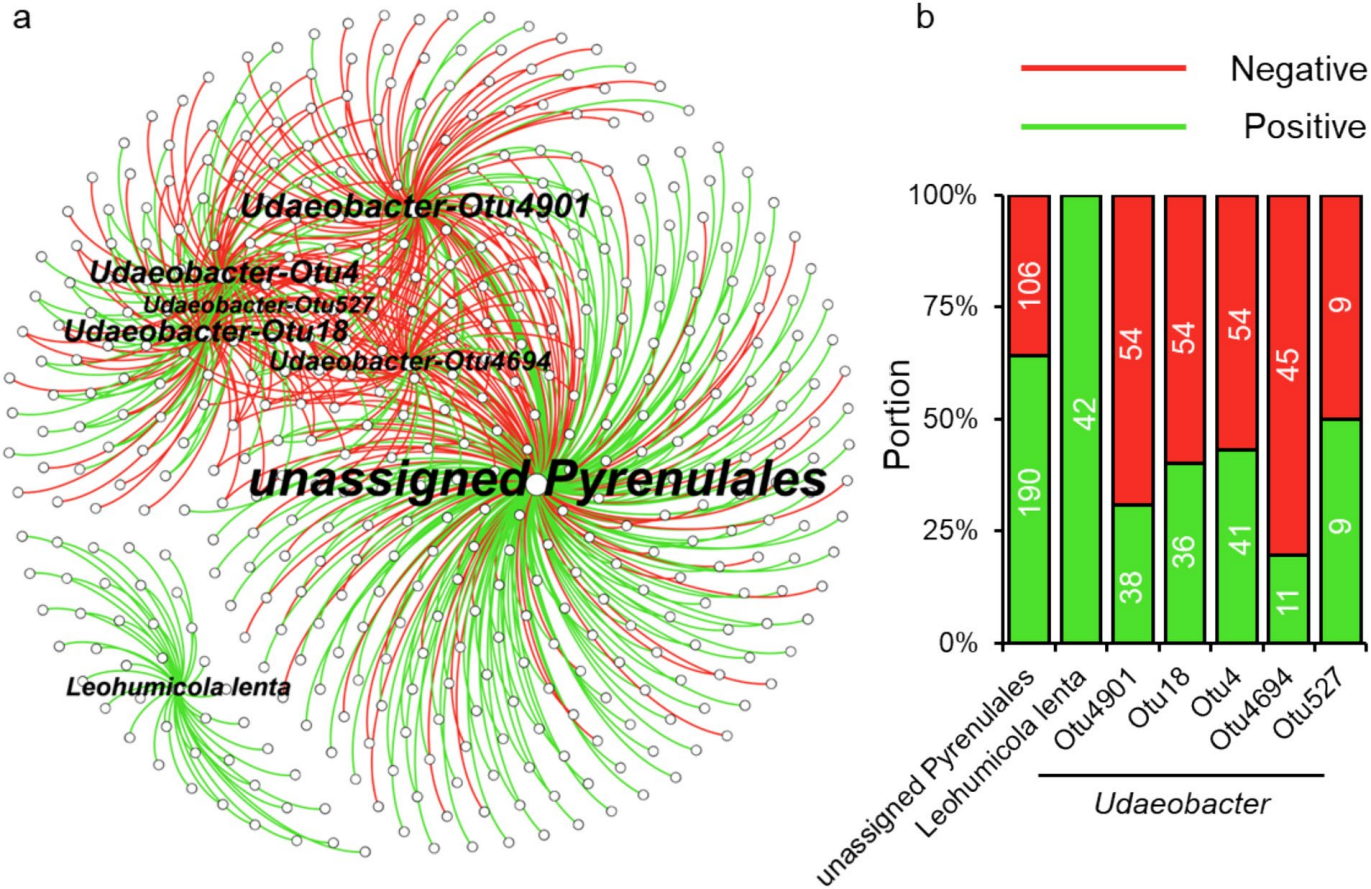
Linkage of dominant featured microbial phylotypes with others in the co-existence network. **(A)** Dominant microbial features (*Udaeobacter*, uncultured *Pyrenulales*, and *Leohumicola lenta*) and their related phylotypes in the network. **(B)** Portions of their interaction types (positive or negative edges) with other microbial phylotypes in the network.

We constructed a microbial co-existence network comprising 2,134 nodes and 31,986 edges to explore the contribution of featured microbial phylotypes to microbial community structure. The network was predominantly composed of bacteria, accounting for 76.76% of the total nodes (Figure 5A). It showed a well-modular structure with bacteria and fungi tending to form their respective modules (Figure 5B). Among the 201 featured microbial phylotypes identified above, only 22 were absent from the network, primarily due to rare species filtering *a-prior* to network calculation. In other words, the majority (90%) of featured microbial phylotypes are significantly related to other microbes. By contrast, the overall microbial community displayed a significantly lower correlation ratio (only 22% of the overall microbial phylotypes were included in the network). Featured microbial phylotypes represented only 0.33% of the total number of nodes in the entire network but were directly related to 453 species, accounting for 21.2% of the total network nodes. We further extracted the phylotypes relating to the three dominant microbial features *Udaeobacter*, unassigned *Pyrenulales*, and *Leohumicola lenta*, into a subnetwork (Figure 6A). The unassigned *Pyrenulales* related to most nodes, while *Leohumicola lenta* formed a separate module. Both unassigned *Pyrenulales* and *Leohumicola lenta* were mainly positively correlated with other microbes, especially the latter whose all edges with others were positive (Figure 6B). By contrast, all featured *Udaeobacter* phylotypes were more negatively than positively correlated with other related phylotypes.

## 4 Discussion

### 4.1 Stoichiometric characteristics and its effects on plant–microbiome interactions in P-deficient acidic soils

A growing number of studies demonstrated that P-deficient acidic soils are crucial shelter for the survival of endangered plants (Wassen et al., 2005; Mao et al., 2021; Zuo et al., 2022); consequently, N/P deposition significantly threatens global biodiversity (Bobbink et al., 2010). Given the pivotal roles of microorganisms in C/N/P biogeochemical cycling and plant’s environmental adaptation, unraveling their relationships with soil stoichiometry under P-deficient conditions would be crucial for the conservation of endangered plants.

In this study, the relationship between microorganisms and stoichiometry of P-deficient soil can be interpreted from two aspects. On one hand, on the community level, the overall bacterial/fungal richness shows an increasing trend as N:P increases, indicating a decrease in microbial diversity under the P deposition threat, and bacteria are more responsive than fungi (Figure 1). This suggests that the microbiome of plants grown in P-deficient soils might develop similar low-P tolerance traits as their host plants. In other words, P deposition, when leading to a reduction in aboveground biodiversity, might also decrease the belowground biodiversity associated with endangered plants. This is against the reports that an increase in P had no effect (Nielsen et al., 2015) or positive effect (Wu et al., 2019) on bulk soil microbial diversity, highlighting the sensitivity of endangered plant microbiomes to global changes. Compared with fungi, bacteria are more sensitive to N rather than P, preferring a moderate C: N (approximately 14); its diversity decreases when N decreases (C: N increases). This is consistent with a collective of findings that bacteria are more responsive to soil P than fungi (Chen et al., 2022; Ling et al., 2017; Yang et al., 2020).

On the other hand, the featured microbial phylotypes had different responses to soil stoichiometry under P-deficient conditions. The top abundant featured microbial phylotypes of *F. danxiaensis*, *Udaeobacter* spp., and unassigned *Pyrenulales* responded similarly to soil stoichiometric characteristics. Their relative abundance decreased with increasing C:N and increased with increasing N:P but showed no response to the C:P ratio (Figure 4). Furthermore, their abundance increases with increasing N availability. These results support the importance of nitrogen limitation for soil microbial activities in P-deficient soils (Zhang et al., 2022). Indeed, nitrogen can become more critical for microbes when phosphorus is limited. In highly weathered soils where phosphorus tends to be bound to iron or aluminum sesquioxides, microbial phosphorus limitation may be common; in such cases, nitrogen availability becomes crucial for microbial growth and function (Liu et al., 2015). While, another featured microbial phylotype *Leohumicola lenta* exhibited no response to C:N, N:P, or C:P. Instead, it exhibited a hump relationship with pH, preferring a pH of approximately 5.5. This species was positively related to all its relevant microorganisms (Figure 6), indicating that it adopted a different ecological strategy from the other two when composing the microbiome of *F. danxiaensis*. Despite the higher abundance of Gammaproteobacteria in the rhizosphere of the reference tree *P. massoniana* (Figure 2), the soil total nitrogen (TN) was significantly lower than in *F. danxiaensis rhizosphere* (Supplementary Figure S1). Given the non-significant difference in total P, and that the sample sites are natural forests (which means no direct N fertilization deployed), such difference in TN explained the difference in N:P between the two trees. This indicates that the biological N-fixation (at least from the Proteobacteria group) is important for the non-endangered plant *P. massoniana* but might not contribute to the adaptation of endangered plant *F. danxiaensis* to P-deficient habitat.

### 4.2 Rhizosphere as the hotspot for *F. Danxiaensis* recruiting its microbiome in P-deficient acidic soil

*F. danxiaensis*, the typical endangered plant species exclusively endemic to Mt. Danxia, demonstrated a remarkable tolerance to the extremely oligotrophic acidic soils there (Chen et al., 2014). The overall soil pH on Mt. Danxia is 4.47 ± 0.29, similar to that of the reference species *P. massoniana* whose rhizosphere soil had a pH of 4.44. However, *F. danxiaensis* held a significantly higher soil pH (5.67) in the rhizosphere (Figure 1), recruiting microbiomes that are preferable to this unique habitat. Our study revealed a higher soil bacterial/fungal diversity in *F. danxiaensis* rhizosphere soils than in *P. massoniana* rhizosphere (Figure 1). This finding echoes the established correlation between pH levels and soil biodiversity, where higher pH levels (below 7) typically support greater microbial diversity (Fierer and Jackson, 2006). Soil pH plays a predominant role in shaping the microbial community, as it can affect the balance of nutrients in the soil, thereby impacting soil stoichiometry. However, it should be noted that the pH–diversity relationship in the rhizosphere does not peak at 7 but at 5.75 (Figure 1). This underscores the specificity of the rhizosphere as a hotspot for functional microbiome recruitment in P-deficient acidic soil. In soils with low phosphorus availability, plants can alter their root architecture and exudate composition in the rhizosphere to attract phosphorus-solubilizing microorganisms (Pantigoso et al., 2023). These microbes release enzymes that convert insoluble phosphorus into bioavailable forms, thereby changing the P uptake and soil stoichiometry and thereafter the interactions of plants with other microbes (Cheng et al., 2023).

The featured phylotype *Udaeobacter* spp. in *F. danxiaensis* microbiome might hold significant ecological implications for the population establishment of *F. danxiaensis*, particularly given that nearly half of the *Udaeobacter* phylotypes are exclusively found in the rhizosphere of *F. danxiaensis* (Figure 3). *Udaeobacter* is a recently established small-genome Verrucomicrobia cluster ubiquitous in global grassland and forest soils (Brewer et al., 2016). It has been revealed that this globally abundant verrucomicrobial clade shows strong preferences over acidic soil between 4.7 and 5.2 (Willms et al., 2021); while in the case of *F. danxiaensis* rhizosphere, soil pH ranged from 4.2 to 6.5 and *Udaeobacter* showed higher abundance toward 6.5 (Figure 4). Currently, knowledge of the ecological significance of *Udaeobacter* remains limited. It can minimize cellular architecture and sacrifice metabolic versatility for efficiency to become dominant in the soil environment and have the capacity to store surplus carbon as glycogen or starch; it also demonstrated the potential to resistant multiple classes of antibiotics and even utilize nutrients released in antibiotic-driven lysis by other soil microbes (Willms et al., 2020), which probably results in the pattern that it was negatively related majority of its associated phylotypes (Figure 6). The genomic evidence suggests that this small-genome bacteria may be auxotrophic (Brewer et al., 2016), possibly indicating an unknown mutual beneficial relationship, e.g., in the form of metabolic interdependence, with its host *F. danxiaensis*. The other two featured phylotypes unassigned *Pyrenulales* and *Leohumicola lenta* are typical microfungi that coexisted with trees (Mack, 2022); their ecological performance on the host remains unreported. Given the above information, our study emphasizes the importance of understanding plant–microbiome interactions in P-deficient acidic soils for the conservation of the endangered plant *F. danxiaensis*.

The conservation of endangered plants also implies opportunities for the development of microbial germplasm resources in the form of potential plant growth-promoting rhizobacteria (PGPR). For example, the featured phylotypes *Udaeobacter* and *Leohumicola lenta* accumulated in the rhizosphere of *F. danxiaensis* are known to be slow-growing on plates; their higher abundance in the rhizosphere habitat suggests that we can more efficiently obtain responsive pure cultured strains by dissecting the interactions between plants and microbiome.

## 5 Conclusion

Taken together, we presented a novel analysis of the microbiome associated with an endangered plant species *F. danxiaensis*. Our results showed that *F. danxiaensis* recruited its unique microbiome, characterized predominantly by *Udaeobacter* spp., which is distinct from that of the reference plant *P. massoniana.* This featured phylotype is highly adapted to the soil traits of *F. danxiaensis* rhizosphere, including a typical high N:P ratio and increased pH in P-deficient soils. While gammaproteobacterial N-fixers are important for the reference plant, they appeared to play a limited role in the microbiome of the endangered plant. Although the functional significance of microorganisms in endangered plant conservation has not yet been fully understood, this study provided evidence for the hypothesis that microbiomes play pivotal roles in sustaining the population development of endangered plants. Our findings underscore the potential importance of microbiome for conservation efforts, particularly in a scenario of global N and P deposition threatening biodiversity in P-deficient acidic soil.

## Data Availability

The datasets presented in this study can be found in online repositories. The names of the repository/repositories and accession number(s) can be found at: https://www.ebi.ac.uk/ena, PRJEB75382.

## Footnotes

^1^ https://www.r-project.org/

## In memoriam

The authors acknowledge the invaluable contributions of Dr. Guangda Tang, a mentor and friend, who regrettably passed away before the submission of this study. His dedication as a researcher and his insightful contributions greatly enriched this study. Dr. Tang will be deeply missed, and his memory will be cherished.
