# Supplementary material for: Characterizing the microbiome recruited by the endangered plant Firmiana danxiaensis in phosphorus-deficient acidic soil
Source: Front Microbiol. 2025 Jan 15;15:1439446. doi: 10.3389/fmicb.2024.1439446 (PMC11774962; doi:10.3389/fmicb.2024.1439446)

**Figure S1. Soil organic matter (SOM), total nitrogen (TN), and total phosphorus (TP) in the rhizosphere of *F*. *danxiaensis* and P. massoniana.** Stars within each graph indicate significant level (**p< 0.01, ***p<0.001, ns - not significant) according to Tukey’s HSD test.


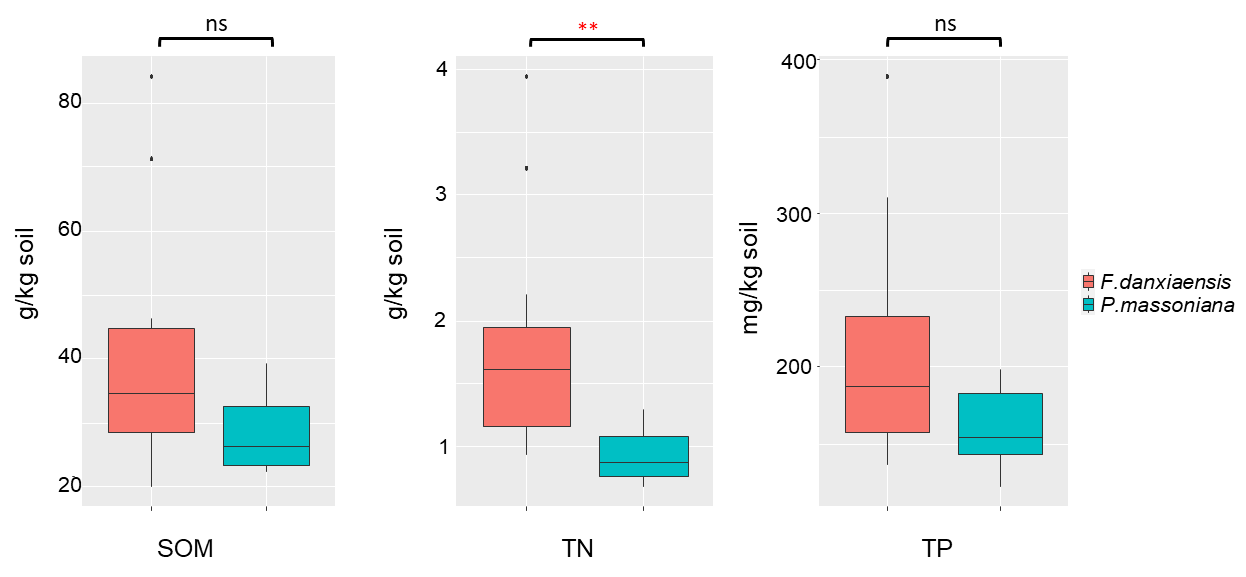


**Figure S2.** **Soil pH and stoichiometry in the rhizosphere of *F*. *danxiaensis* and P. massoniana.** Soil stoichiometry traits include the ratio of carbon to nitrogen (C:N), carbon to phosphorus (C:P) and nitrogen to phosphorus (N:P). Stars within each graph indicate significant level (**p< 0.01, ***p<0.001, ns - not significant) according to Tukey’s HSD test.


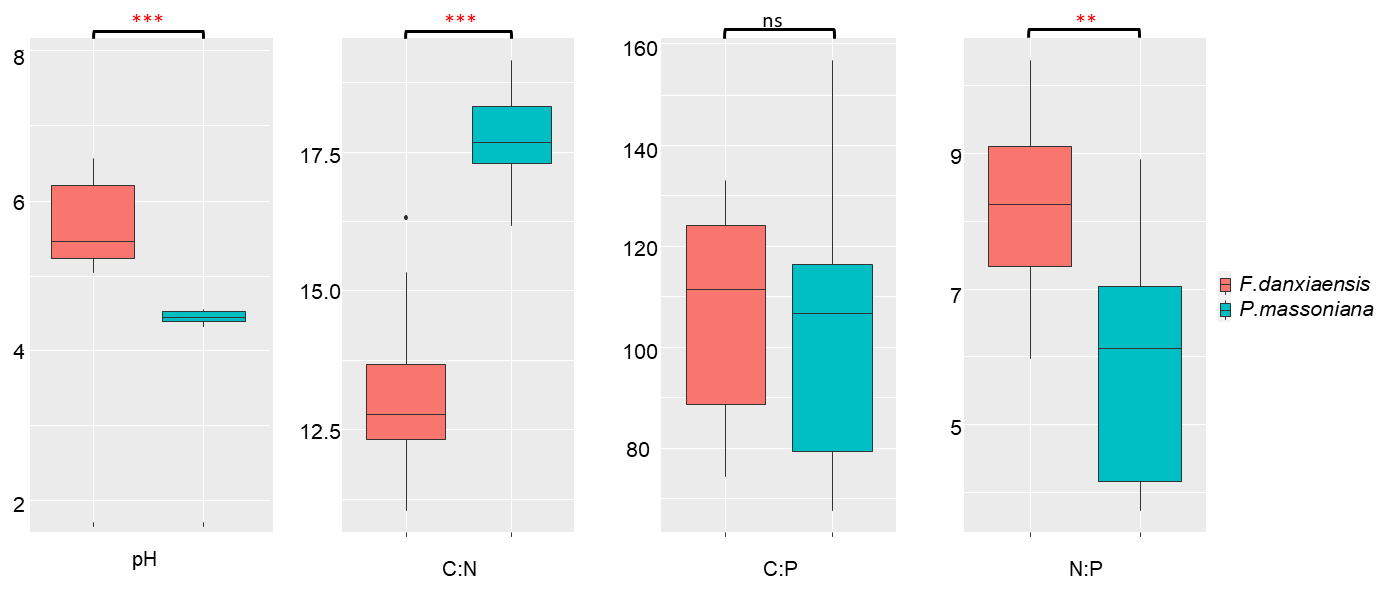


**Figure S3. Effects of sites (a) and host species (b) on the soil properties variation.** The scatter plot was based on the first and second axis of the principal component analysis (PCA), and the significance was tested using PerMANOVA.

**
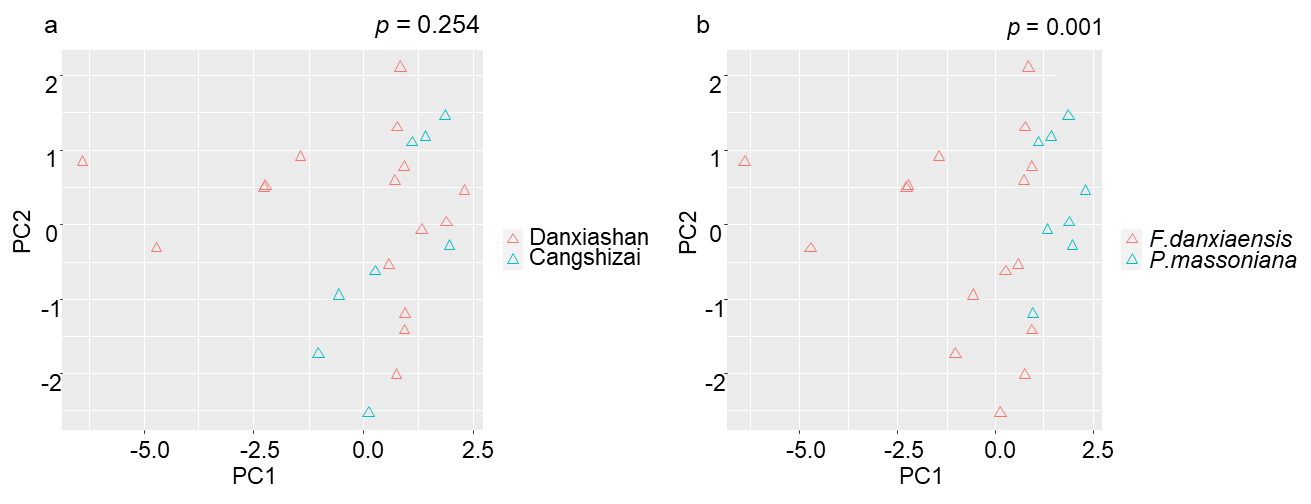
**

**Figure S4. Taxonomic composition of soil bacteria (a) and fungi (b) communities in the rhizosphere of *F. danxiaensis and P. massoniana*** as demonstrated by the relative abundance at phylum and class level, respectively.


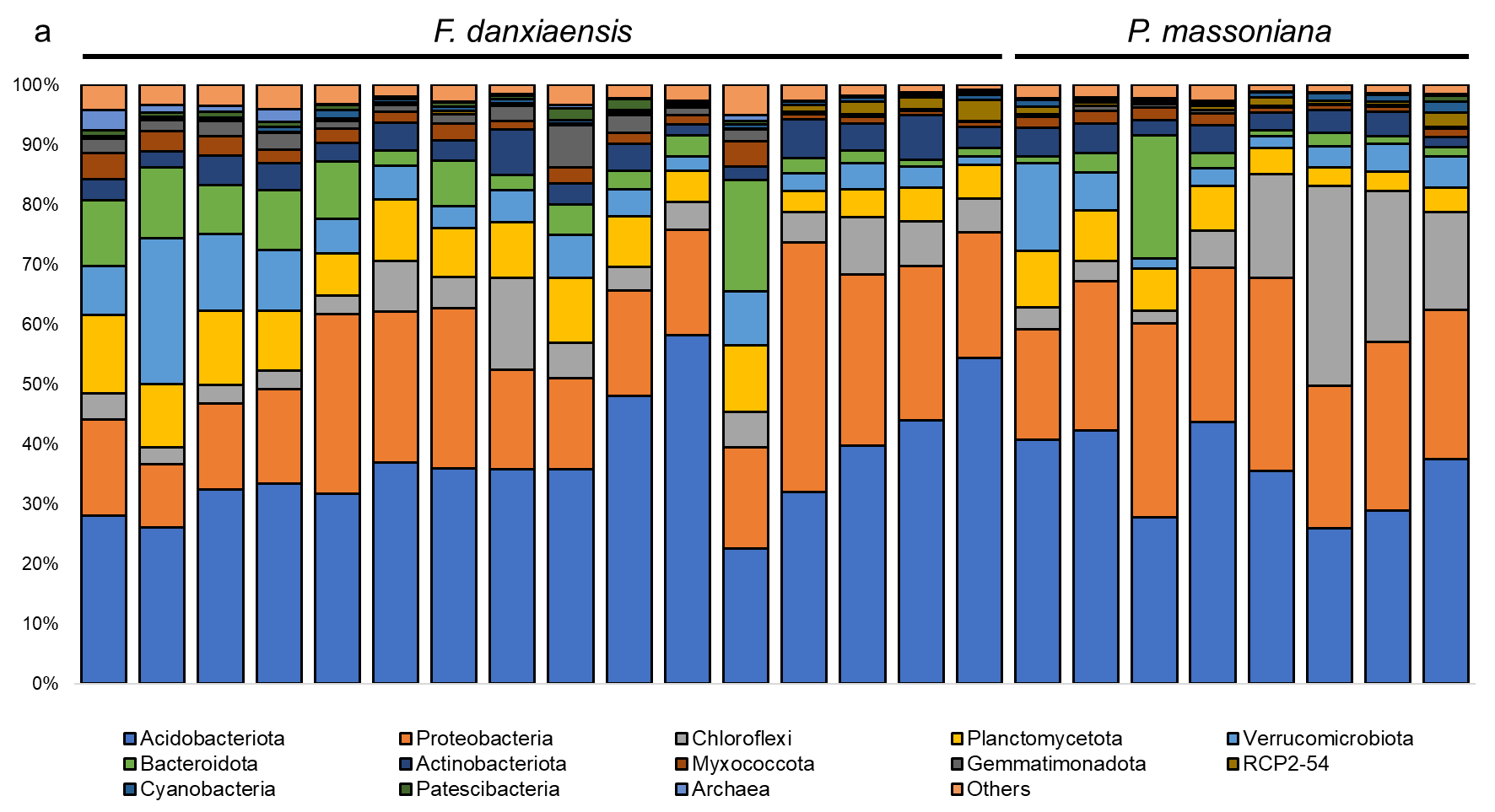


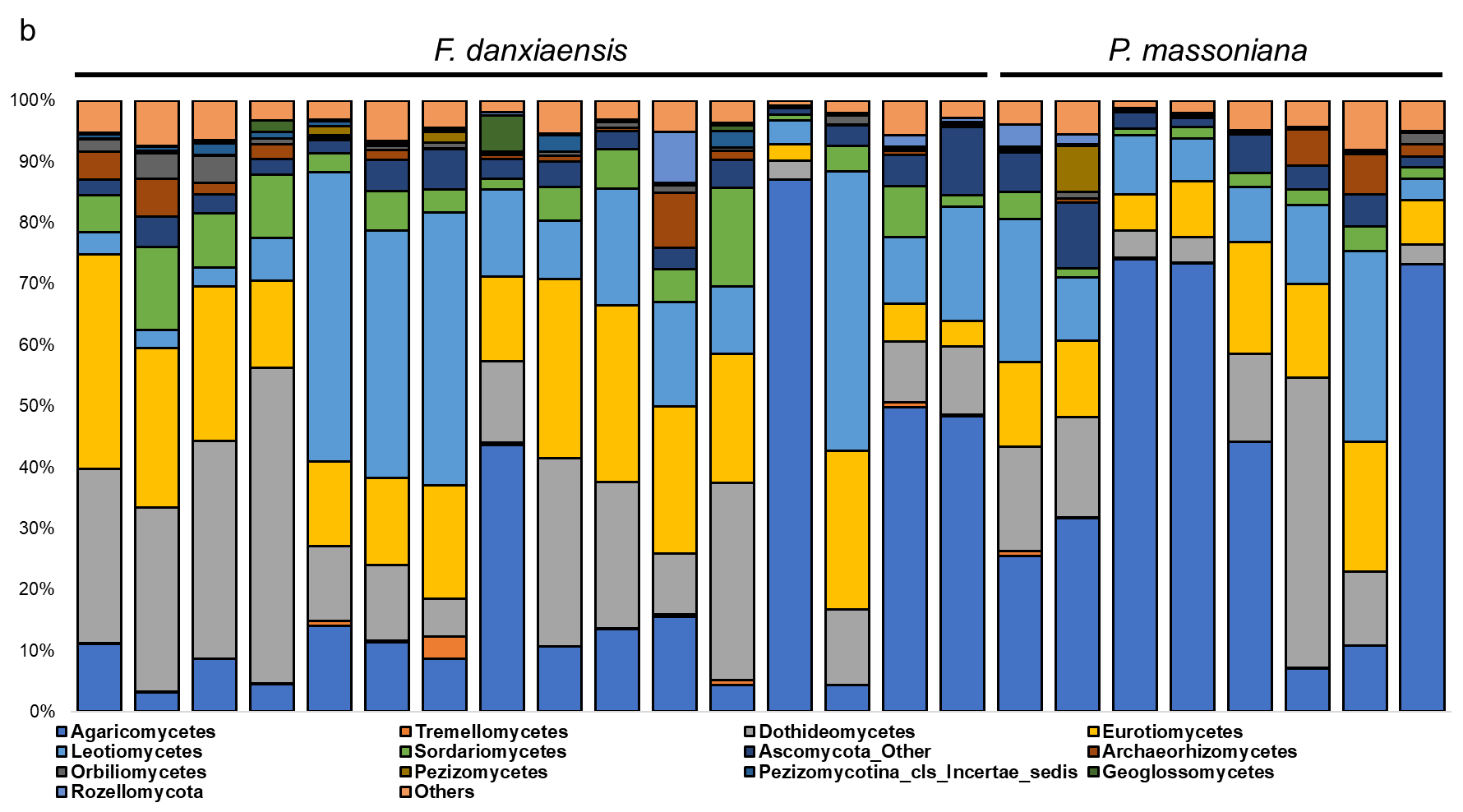

Supplement: Supplementary file 1 [file Data_Sheet_1.docx]
